# Does a pay-for-performance health service model improve overall and rural–urban inequity in vaccination rates? A difference-in-differences analysis from the Gambia

**DOI:** 10.1016/j.jvacx.2022.100206

**Published:** 2022-08-17

**Authors:** Alieu Sowe, Fredinah Namatovu, Bai Cham, Per E. Gustafsson

**Affiliations:** a Department of Epidemiology and Global Health, Umeå University, Umeå, Sweden; b Expanded Program on Immunization, Ministry of Health, Banjul, Gambia; c Shifo Foundation, Stockholm, Sweden; d Medical Research Council Unit The Gambia at the London, School of Hygiene and Tropical Medicine, Bakau, Gambia

**Keywords:** Vaccination inequalities, Vaccination coverage, Results-based financing, Pay-for-performance, Residential inequalities

## Abstract

**Objective:**

To assess whether the implementation of a results-based financing (RBF) project in The Gambia resulted in (1) improved national vaccination coverage (2) higher coverage in intervention than non-intervention areas, and (3) improvement in rural–urban coverage inequality.

**Methods:**

The study used a difference-in-differences design, based on repeated cross-sectional data from The Gambian Demographic and Health Surveys 2013 (N = 1650) and 2020 (N = 1456). Full vaccination (receipt of one BCG, 3 OPV, 3 DTP, and 1 measles-containing vaccine doses) and rural–urban vaccination inequality were our outcome variables. The intervention, RBF, was implemented in 5 of the 7 health regions. Covariates controlled for included child’s sex, child’s birth order number, socioeconomic status, ethnicity, distance from health facility, maternal education, mother’s age group, mother’s marital status, and mother’s work status. Poisson regression with robust variance was used to estimate whether coverage changed, and difference-in-differences and difference-in-differences-in-differences were used to ‘assess differences in vaccination coverage change and change in inequalities, respectively.

**Results:**

Total crude full vaccination coverage in The Gambia was 76% in 2013 and 84.6% in 2020. Overall vaccination significantly increased by 16% (95% CI: 9% to 24%) in 2020 compared to 2013, but with a smaller increase in intervention relative to non-intervention areas [PRR 0.88 (CI: 0.78–0.99)]. Rural-urban inequality in vaccination coverage decreased more – by 13% [0.87 (0.78–0.98)] – in RBF than non-RBF regions.

**Conclusion:**

Vaccination coverage improved over the study period though we have no evidence to ascribe the coverage gains to the RBF intervention. However, our study suggests that the RBF project has contributed to reducing rural–urban inequalities in the regions it was implemented.

## Highlights

• Vaccination coverage increased by 16 percentage points in The Gambia during the period 2013–2020.
• Interestingly, non-intervention regions experienced slightly (12%) higher increments in coverage than the pay-for-performance intervention regions.
• Rural-favoured vaccination inequalities decreased more in the pay-for-performance group in comparison with the control group.

## 1 Introduction

Vaccination is one of the most effective public health interventions for disease prevention and control. Following the establishment of the Expanded Program on Immunization (EPI) in 1974 by the World Health Organization [1], an estimated 2–3 million potential deaths due to vaccine-preventable diseases have been averted annually. All countries are committed to improving their national vaccination coverage, consequently reducing the burden of vaccine-preventable diseases, and good progress has been registered to that effect [2].Periodic equity-focused strategic documents have also been developed to guide global vaccination actors and countries, such as the Global Vaccine Action Plan 2011–2020 [3] and its successor, the Immunization Agenda 2030 [4], [5]. Despite these efforts, vaccination coverage and equity challenges remain at both the global and country levels. Over the last decade, the average global vaccination coverage only increased by 5% and has plateaued at around 86% since 2016, leaving about 20 million children un- or under-vaccinated, with over 8 million of them living in Africa [6]. This highlights the urgent need to improve vaccination coverage and equity between and within countries to achieve universal vaccination.

This mixed situation of progress and challenges is well illustrated in The Gambian context. In the beginning, The Gambia Expanded Program on Immunization targeted six diseases (tuberculosis, tetanus, diphtheria, poliomyelitis, pertussis, and measles), gradually adding new vaccines leading to substantial reductions in morbidity and mortality attributable to vaccination targeted diseases.

Despite these areas of success, the country's vaccination program faces challenges in both overall coverage and equity in coverage. Administrative national coverage for individual vaccine doses has mainly decreased or remained the same annually since 2013 [7], similar to the global trend in the last decade. Full vaccination coverage, a composite coverage measure for the six primary immunization targeted diseases, peaked at 87.4% in 2010 and has remained low since then [8], [9], [10], [11], [12]. The coverage challenge is further marred with prominent and persistent socioeconomic and geographic inequalities [13]. However, the direction of these inequalities has unexpectedly been unfavourable to population groups conventionally considered socially privileged. For example, the 2013 Demographic and Health Survey (DHS) reported 16.8 percentage points higher coverage in rural than urban areas and 24 percentage points greater coverage in the poorest than richest household wealth quintile [11]. The paradoxical 'reverse' rural–urban vaccination inequality has been observed and has persisted since at least the mid-1990s [8]. A study that attempted to identify explanatory factors for this disparity attributed most of the variation to material conditions such as household wealth quintile and maternal occupation [13]. The study reported that vaccination coverage was higher among the poorest household quintile and mothers working in agriculture, which are more frequent in rural than urban areas. Another study pointed out weaker social networks for vaccination in urban areas, long waiting times at health facilities, and potential social exclusion of immigrants as factors demotivating vaccination in urban areas [14].

With support from the World Bank, The Gambia has in 2014 implemented a pay for performance project locally known as RBF (results-based financing), which aims to improve maternal and child health service delivery and uptake [15]. It was piloted in one health region and later scaled up to four additional regions, thereby covering five of the seven health regions in the country. Health facilities and communities get remunerated based on the quantity and quality of predetermined indicators, including vaccination-related indicators at health facilities [16].

Our study aimed to track vaccination coverage and equity in The Gambia following the implementation of the RBF project. We first assessed whether there was a change in vaccination coverage after implementing the project; second, we evaluate whether the change in vaccination coverage differed between the regions in which the project was implemented and other regions; and third, we determined if there was a difference in rural–urban vaccination disparity between project and non-project areas.

## 2 Materials and methods

### 2.1 Study design and population

The study employed a difference-in-differences design, based on secondary repeated cross-sectional data from the children's datasets of The Gambia DHS conducted in 2013 [11] and 2019/2020 [17]. A binary variable, *Results-based financing* (RBF) *status,* was used to differentiate regions where RBF was implemented from non-RBF regions, with RBF regions coded as 1 (k = 5 regions) and non-RBF regions 0 (k = 3 regions). Pre-and post-RBF intervention periods, *RBF year* variable, were denoted by 0 (year = 2013) and 1 (year = 2019/2020) respectively. This resulted in four comparison groups for the difference-in-differences design, i.e., preintervention-RBF regions, preintervention-non-RBF regions, postintervention-RBF regions, and postintervention-non-RBF regions.

For simplicity, the second DHS, 2019/2020, will be referred to as just 2020. Data collection for the 2020 survey was conducted from 21st November 2019 to 30th March 2020, before the first Gambian Covid 19 wave emerged in full (The first Covid-19 case in The Gambia was reported Mach 10th, 2020).

A stratified two-stage selection procedure was implemented to select the DHS samples. The 2013 and 2020 DHS waves used enumeration areas from the 2003 and 2013 national censuses as sampling frames. The Gambia is divided into two municipalities and six local government areas (LGA), with the two municipalities considered entirely urban. The health system is divided into seven health regions. The six LGAs were stratified into rural–urban strata, resulting in 14 sampling strata. For the first stage, the areas within each sampling stratum were sorted by lower administrative units (districts and wards) to achieve implicit stratification. The average number of households per enumeration area was 68. Then a predetermined number of areas was then independently selected from each stratum using probability proportional to the estimated area size selection procedure. A total of 281 areas was selected in each survey [11], [17]. Following this, household listing exercises were conducted to update the number of households in the selected areas. Then came the second selection stage. In this stage, 25 households were selected from each area using equal probability systematic sampling [11], [17].

All women aged 15–49 years resident in selected households or who spent the night before the survey in the selected household were eligible survey respondents regardless of their residence status in the area. The number of eligible women interviewed, and response rates for the 2013 DHS and 2020 DHS are 10,233 and 11,865 and 90.7% and 95.1%, respectively. Relevant information, including the vaccination history of each child under five years born to an interviewed woman, was collected. For this study, all children 12–23 months were selected for inclusion because all children in this age cohort are expected to have received all the recommended basic vaccine doses [18]. There are 1660 children aged 12–23 months in the 2013 DHS [11] and 1456 in the 2020 DHS [17]. The proportion of children 12–23 months with vaccination cards was high in both surveys – 90.2% in 2013 and 93.2% in 2020 – with higher proportions in rural than urban areas.

### 2.2 Results-based financing intervention in the Gambia

The RBF project was scaled up from 1 to 5 regions in 2016. The first DHS was conducted in 2013 [11], a year before the project's start, and the second one in 2019/2020, three years after the project was extended to four more regions. The project buys predetermined quantity and quality indicators from health facilities. Quantity indicators are purchased per service delivered, whilst quality indicators are paid based on composite percentage scores attained by health facilities following a quality monitoring checklist. Vaccination performance is remunerated under the quality indicators category. Sixty percent of payments made to health facilities is earmarked for service delivery improvement, and the remaining 40% is shared amongst staff. Vaccination aspects monitored in the quality checklist include valid (doses which are age (and interval in case of multidose vaccines) appropriate doses administered; dropout rate; availability of recording, reporting, and monitoring tools; availability of job aids and adherence to standard operating procedures; availability, functionality, and maintenance of vaccine storage equipment; availability and storage of vaccines and all related supplies and; vaccine wastage [16]. These components of the immunization program are undoubtedly essential for the effective delivery of vaccination services.

### 2.3 Variables

#### 2.3.1 Outcome variables

Full vaccination (aim 1 and 2) was defined as children 12–23 months who had received one dose of Bacillus Calmette Guerin vaccine, one dose of a measles-containing vaccine, three doses of the oral polio vaccine, and three doses of a diphtheria, pertussis, and tetanus-containing vaccine. Both vaccination history by card and recall were included.

Rural-urban vaccination coverage inequality (aim 3) was operationalized as the disparity in full vaccination coverage between rural and urban areas in RBF regions and rural and urban areas in the non-RBF regions. The Gambia Bureau of Statistics’ designation of census areas as rural or urban was used as this was the rural–urban stratification used by the DHS surveys.

#### 2.3.2 Covariates

Covariates were identified based on priori and their availability in the DHS data sets.

*Child's birth order number* was recoded into 1, 2&3, 4&5, and 6 or above, while her/his *sex* was considered as male or female. *Mothers' ages* were grouped into 15–24 years, 25–29 years, 30–34 years, and 35–49 years and their *marital statuses* were defined as currently married or not currently married. Ethnicity was grouped as Wolof, Mandinka, Fula, others, and non-Gambians.

*Household socioeconomic status* was generated from the household wealth quintile variable in the DHS data set. The household wealth quintile is a composite measure of relative household wealth created through principal component analysis using household ownership or access to materials including televisions and bicycles; materials used for housing construction; and types of water access and sanitation facilities [19]. We recategorized quintiles of household socioeconomic status into three groups of rich, middle, or poor by merging the richer and richest quintiles into rich and the poorer and poorest quintiles into to poor while leaving the middle category unchanged. A child's mother’s *work status* was considered as either not working or informal work, or formal work, and their *education* was grouped as no education, primary or secondary and above.

*Distance to a health facility* when in need of healthcare services was classified as a big problem or not a big problem based on the response of the caregiver interviewed.

### 2.4 Approach to data analysis

All analyses in this paper were performed using Stata software version 17 [20]. The “svy” command in Stata for survey data analysis was utilized in all analysis to account for the complex design (survey weights, clustering, and stratification) of the surveys. The analytical code is attached as supplemental material 1.

#### 2.4.1 Descriptive analysis

In the first set of analysis, bivariate analyses were performed to estimate the frequency of full vaccination across exposure variables. We then estimated rural, urban, and total full vaccination coverage for the RBF intervention group, the control group, and The Gambia pre- (2013) and post-RBF intervention (2020).

#### 2.4.2 Main analysis

Following the set of analysis corresponding to the first aim, we utilized Poisson regression with robust variance to evaluate the crude (bivariate) and adjusted (including all covariates) relative change in overall vaccination coverage from 2013 to 2020. To address the second aim, we then evaluated whether there is a difference in changes in full vaccination coverage between RBF and non-RBF intervention sites using difference-in-differences (DiD) analysis in crude and adjusted analyses. Finally, corresponding to the third aim, we assessed the effects of RBF implementation on rural–urban disparities in vaccination coverage between intervention and control areas through a difference-in-difference-in-difference (DiDiD) approach in crude and adjusted analyses. We operationalized the DiD and DiDiD by generating variables that are equal to the product of the respective variables of interest. We used RBF implementation status and RBF year variables for the DiD and added residence to these two variables for the DiDiD analysis.

We reported crude and adjusted Prevalence Rate Ratios (PRRs) and their 95% confidence intervals (CIs). Where we report p-values, we take statistical significance to be p ≤ 0.05.

#### 2.4.3 Sensitivity analysis

Since there is a difference in vaccination card retention rates between urban and rural areas and a difference in vaccination coverage among children with and those without cards, we conducted a sensitivity analysis on the potential effects of recall bias on our main results. We excluded children whose vaccination histories were obtained through caregivers recall and repeated our analysis, then compared the results with those including all eligible children (N = 3116). Please see the supplemental material attached.

### 2.5 Ethical considerations

Demographic and Health Surveys are standard nationally representative household surveys conducted in developing countries to shed light on demographic and health trends across several dimensions. They are ethically cleared by ICF Institutional Review Board (IRB) and usually by IRBs of countries conducting the surveys [21]. Fieldwork for the two surveys in The Gambia was conducted by trained data collectors who interviewed respondents only after obtaining their informed consent. Anonymized DHS datasets are publicly available via the DHS program website https://dhsprogram.com/data/available-datasets.cfm [22].

## 3 Results

### 3.1 Descriptive analysis

Table 1 below shows the background characteristics of survey respondents. About 200 less participants were surveyed in 2020 than 2013. The numbers of respondents in non-RBF and RBF intervention areas were similar (859 vs 801) in 2013, unlike 2020, in which fewer children were sampled in RBF regions. Consistent with the characteristics of the intervention and control groups, non-RBF regions were mainly urban while RBF intervention regions were to a greater extent rural. The number of non-intervention ruralresidents in the 2020 DHS (n = 29) is small in comparison with that of 2013 (n = 163). In terms of household socioeconomic status, there is no major difference in the distribution of children in the different categories in the two survey periods by RBF implementation except for the middle category. The proportions of children in the middle socioeconomic households of non-RBF and RBF areas in 2013 changed from 36.4% and 63.6% to 66.2% and 33.8%, respectively, in 2020.

**Table 1:** Weighted distribution of participants across covariates in RBF and non-RBF intervention areas in 2013 and 2020 (row % by survey year).

Variable	DHS 2013 (N = 1660)		DHS 2020 (N = 1456)	
	Non-RBF n = 859	RBF n = 801	Non-RBF n = 859	RBF n = 597
*Residence*				
Urban	696 (89.7%)	80 (10.3%)	830 (86.1%)	134 (13.9%)
Rural	163 (18.4%)	721 (81.6%)	29 (5.8%)	463 (94.2%)

*Household socioeconomic status*				
Poor	204 (29.0%)	499 (71.0%)	180 (29.2%)	437 (70.8%)
Middle	130 (36.4%)	227 (63.6%)	209 (66.2%)	107 (33.8%)
Rich	525 (87.5%)	75 (12.5%)	469 (89.7%)	54 (10.3%)

*Mother's marital status*				
Not currently married	68 (70.1%)	29 (29.9%)	85 (82.5%)	18 (17.5%)
Currently married	791 (50.6%)	772 (49.4%)	773 (57.2%)	579 (42.8%)

*Child's sex*				
Male	437 (51.2%)	417 (48.8%)	443 (59.1%)	306 (40.9%)
Female	422 (52.3%)	384 (47.7%)	415 (58.8%)	291 (41.2%)

*Child's ethnicity*				
Mandinka	322 (54.8%)	265 (45.2%)	305 (65.5%)	160 (34.5%)
Wolof	131 (38.4%)	211 (61.6%)	104 (35.1%)	191 (64.9%)
Fula	137 (34.4%)	260 (65.6%)	119 (44.1%)	152 (55.9%)
Others	162 (87.5%)	23 (12.5%)	174 (91.4%)	16 (8.6%)
Non-Gambia	107 (72.2%)	41 (27.8%)	156 (66.7%)	78 (33.3%)

*Maternal education*				
No education	357 (37.9%)	586 (62.1%)	267 (42.7%)	358 (57.3%)
Primary education	140 (56.4%)	108 (43.6%)	187 (61.7%)	116 (38.3%)
Secondary education	362 (77.1%)	107 (22.9%)	404 (76.7%)	123 (23.3%)

*Mother's work status*				
Not working	445 (60.5%)	290 (39.5%)	360 (66.3%)	183 (33.7%)
Working	414 (44.8%)	511 (55.2%)	498 (54.6%)	414 (45.4%)

*Distance to a health facility*				
Not a big problem	698 (61.6%)	436 (38.4%)	718 (65.6%)	377 (34.4%)
Big problem	161 (30.6%)	365 (69.4%)	140 (38.9%)	220 (61.1%)

*Mother's age group*				
15–24 years	238 (46.6%)	273 (53.4%)	196 (54.6%)	163 (45.4%)
25–29 years	283 (61.3%)	179 (38.7%)	320 (64.5%)	176 (35.6%)
30–34 years	169 (49.3%)	174 (50.7%)	175 (57.8%)	128 (42.2%)
35–49 years	169 (49.0%)	176 (51.0%)	168 (56.4%)	130 (43.6%)

*Child's birth order number*				
1	191 (52.7%)	172 (47.3%)	188 (62.3%)	114 (37.7%)
2 & 3	340 (59.4%)	233 (40.6%)	358 (65.4%)	190 (34.6%)
4 & 5	165 (46.4%)	191 (53.6%)	181 (54.1%)	154 (46.0%)
6 & above	162 (44.2%)	205 (55.8%)	131 (48.4%)	140 (51.6%)

In Table 2 below, total crude full vaccination coverage in The Gambia was 76% in 2013 and 84.6% in 2020. As in the general population, coverage is higher in rural than urban areas in the intervention and control groups. Baseline coverage is higher in the intervention (82.9%) than in the control group (69.6%), but post-intervention coverages between the two groups are similar.

**Table 2:** Weighted full vaccination coverage in The Gambia 2013–2020.

Variable	Pre-intervention coverage (year = 2013)	Post-intervention coverage (year = 2020)	Average coverage
*Intervention group*			
Urban	69.8% (59.0%–78.8%)	78.0% (69.4%–84.6%)	74.9% (68.3%–80.6%)
Rural	84.4% (81.1%–87.1%)	89.8% (87.0%–92.0%)	86.5% (84.1%–88.5%)
Total	82.9% (79.6%–85.8%)	87.1% (84.2%–89.6%)	84.7% (82.5%–86.7%)

*Control group*			
Urban	66.8% (57.6%–74.9%)	82.3% (77.4%–86.3%)	75.2% (69.8%–80.0%)
Rural	81.6% (72.8%–88.1%)	100.0% (. -.)	84.4% (76.2%–90.1%)
Total	69.6% (61.9%–76.4%)	82.9% (78.1%–86.7%)	78.4% (74.8%–81.5%)

*The Gambia*			
Urban	67.1% (58.9%–74.4%)	81.7% (77.4%–85.3%)	75.2% (70.4%–79.4%)
Rural	83.9% (80.7%–86.6%)	90.4% (87.6%–92.5%)	86.2% (83.9%–88.1%)
Total	76.0% (71.4%–80.1%)	84.6% (81.7%–87.1%)	80.0% (77.1%–82.7%)

### 3.2 Change in coverage 2013–2020

Full vaccination coverage in The Gambia increased during the period 2013–2020, as can be deduced from Table 3 below. Full vaccination was 11% (CI: 4%–19%) higher among children surveyed in 2020 than those surveyed in 2013 in crude analyses, which increased to 16% (CI: 9%–24%) after consideration of covariates. In the adjusted model, residence, ethnicity, and child's birth order number were associated with full vaccination. Children resident in rural areas were 20% more commonly vaccinated than their urban counterparts. Vaccination was less frequent among the Fula ethnic group and non-Gambians than the Mandinka ethnic group. Children belonging to the second or third birth order category were 12% more frequently vaccinated than those in the first birth order category. Marital status, education, work status, age group, sex or perceived distance to a health center were not significantly associated with full vaccination.

**Table 3:** Weighted prevalence rate ratios of the change in vaccination coverage in The Gambia 2013 – 2020.

Variable	Crude model	Adjusted model
*Year*		
2013	Reference	Reference
2020	**1.11 (1.04**–**1.19)**	**1.16 (1.09**–**1.24)**

*Residence*		
Urban	Reference	Reference
Rural	**1.15 (1.07**–**1.22)**	**1.20 (1.10**–**1.30)**

*Household socioeconomic status*		
Poor	Reference	Reference
Middle	0.97 (0.92–1.02)	1.02 (0.96–1.08)
Rich	**0.91 (0.84**–**0.98)**	1.03 (0.95–1.12)

*Mother's marital status*		
Not currently married		
Currently married	1.05 (0.95–1.16)	0.99 (0.88–1.11)

*Child's sex*		
Male	Reference	Reference
Female	0.96 (0.92–1.01)	0.97 (0.92–1.01)

*Child's ethnicity*		
Mandinka	Reference	Reference
Wolof	0.97 (0.90–1.04)	0.95 (0.88–1.01)
Fula	**0.90 (0.85**–**0.96)**	**0.89 (0.83**–**0.94)**
Others	0.95 (0.87–1.03)	0.97 (0.90–1.05)
Non-Gambian	**0.76 (0.67**–**0.86)**	**0.76 (0.68**–**0.86)**

*Maternal education*		
Not literate	Reference	Reference
Primary education	1.03 (0.97–1.09)	1.02 (0.96–1.08)
Secondary education	0.97 (0.90–1.04)	0.99 (0.91–1.07)

*Mother's work status*		
Not working	Reference	Reference
Working	**1.09 (1.03**–**1.16)**	1.02 (0.97–1.08)

*Distance to a health facility*		
Not a big problem	Reference	Reference
Big problem	1.03 (0.97–1.08)	1.00 (0.95–1.05)

*Mother's age group*		
15–24 years	Reference	Reference
25–29 years	1.03 (0.97–1.10)	0.98 (0.91–1.06)
30–34 years	1.04 (0.97–1.11)	0.99 (0.91–1.08)
35–49 years	**1.09 (1.02**–**1.17)**	1.06 (0.97–1.16)

*Child's birth order number*		
1	Reference	Reference
2 & 3	**1.13 (1.04**–**1.24)**	**1.12 (1.03**–**1.23)**
4 & 5	**1.12 (1.02**–**1.22)**	1.07 (0.97–1.19)
6 & above	**1.14 (1.04**–**1.24)**	1.05 (0.94–1.17)

The inferences concerning change in vaccination coverage from 2013 to 2020 remained when we restricted the analysis to only children with vaccination cards (supplemental material 1), but with slightly lower point estimates [crude PRR 1.09 (CI: 1.02–1.15); adjusted PRR 1.12 (CI: 1.06–1.19)] 9% in crude analyses and 12% in adjusted analyses.

### 3.3 Differences in the change in coverage between intervention and control regions

The crude DiD PRR for full vaccination pre- and post-intervention between RBF and non-RBF areas was 0.88 (CI: 0.78–1.00). When adjusted for covariates, the ratio remained the same, 0.88 (CI: 0.78–0.99), but reached statistical significance. This highlights that RBF intervention areas had a lower (12% less) coverage increment during 2013 – 2020 compared to the non-intervention areas. Full analyses are attached as supplemental material 2 Tables 1 and 2.

In sensitivity analyses (supplemental material 3 Tables 3 and 4) excluding children with vaccination history obtained by recall, the crude and adjusted PRRs were weaker and statistically insignificant (Crude PRR = 0.92: CI 0.82–1.03: P value = 0.18; Adjusted PRR = 0.91: CI 0.81–1.01: P value = 0.11).

### 3.4 Rural-urban differences in change in coverage between intervention and control regions

**Fig. 1:**
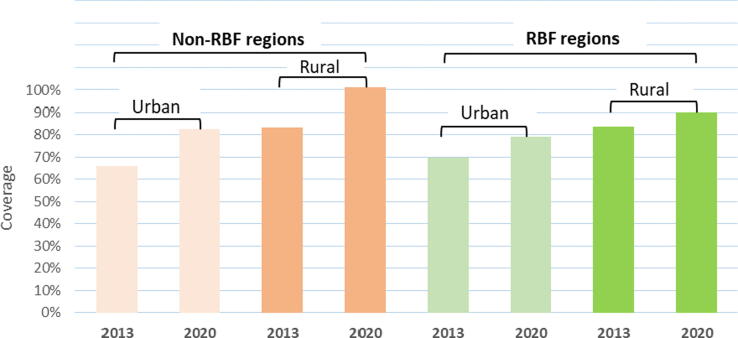
Adjusted ** predicted mean vaccination coverage margins for rural and urban areas in 2013 and 2020 by RBF implementation status. ** Adjusted for household socioeconomic status, mother's marital status, child's sex, child's ethnicity, maternal education, mother's work status, distance to a health facility, mother's age group, and child's birth order number.

The difference in rural–urban coverage between RBF and non-RBF regions was lower in 2020 than in 2013 [PRR = 0.88 (CI: 0.78–1.00)], albeit marginally significant (P value = 0.05). Following adjustment for covariates, the difference-in-differences in rural–urban coverage inequality between RBF and non-RBF regions was reduced by 13% [0.87 (0.78–0.98)], indicating that rural–urban vaccination inequality decreased more in the intervention than control areas in 2020 relative to their 2013 baseline (supplemental material 2 Tables 3 and 4). A graph of the predicted coverage means by group in 2013 – 2020 can be seen in Fig. 1. For this analysis, excluding vaccination history by recall (supplemental material 3 Tables 5 and 6) yielded slightly weaker and insignificant results but in the same direction (Crude PRR = 0.92; P value = 0.15; Adjusted PRR = 0.91; P value = 0.09).

## 4 Discussion

Our study aimed to determine whether vaccination coverage changed in The Gambia following a pay-for-performance intervention and assess how the intervention impacted inequalities in coverage. We found an overall increase in full vaccination coverage over 2013–2020. However, in comparing vaccination rates between the intervention and control group, our results showed a more noticeable improvement among children resident in control rather than intervention areas. Regarding the rural–urban vaccination disparity, our results showed the inequality shrank more post-RBF in RBF implemented than in non-intervention areas. Taken together, our findings showed an overall improvement in vaccination coverage, although we were unable to attribute the change to the implementation of the RBF project as project sites surprisingly displayed a smaller increase in coverage. Notwithstanding, our study also revealed that the difference in rural–urban vaccination improved more in RBF area than in the non-RBF area in 2020.

The pay for performance approach is increasingly becoming popular due to its potential to improve health systems [23] and promote equity in health service utilization [24]. A recent review reported that pay for performance schemes had been implemented in at least 23 African countries [25]. Many studies on the impact of pay-for-performance models on vaccination indicate that these schemes can increase vaccination rates [26], [27], [28]. Nonetheless, there are studies which reported that it had no effect on vaccination [29] and had not improve socioeconomic inequalities in vaccination [30].

Our finding that average national vaccination coverage increased over the study period is encouraging, especially when global vaccination rates seem to stall. For a long time, Gambian women have trusted reproductive and child health clinics through which vaccinations are offered [31]. They even willingly accept vaccine administration routes new to them, such as intranasal administrations [32]. This favourable behaviour of caregivers coupled with the increased support received by the vaccination program such as the Gavi health system and immunization strengthening grant [33] may have contributed to the observed improvements.

It is somewhat unexpected that we found coverage increased more in non-RBF than RBF areas. One would expect that coverage will increase more in the RBF areas. Some studies have shown that pay for performance interventions do not substantially address inequalities unless the disparities are vast or the interventions are designed to tackle underlying disparities [34], [35], [36]. In contrast with our finding, a study in Estonia found that mean vaccination rates were higher among family doctors who participated in a pay for performance quality system than those who did not [27]. The ceiling effect may, in part, explain our findings considering that the RBF regions had higher baseline vaccination rates. Vaccination coverage is much more difficult to improve in areas that have a high already at baseline [37]. Pay-for-performance schemes can increase healthcare service utilization by removing access-related barriers in groups with access challenges [24]. Geographical access to vaccination services is not a significant problem in The Gambia due to regular monthly outreach vaccination sessions conducted in communities far from health facilities. Monthly contact with the health system is sufficient to ensure optimal vaccination of children [38]. Therefore, since the RBF project did not increase vaccination service delivery points in project areas and that vaccine stockout is seldom in the country, the intervention regions did not have much practical access advantage over the non-intervention regions.

Furthermore, the key vaccination-related indicators purchased by the RBF project are part of routinely monitored immunization program indicators. The usually poorer routine immunization performance of non-RBF regions is a common subject of discussion during vaccination-related review meetings in the country. Perhaps, this motivated health workers in non-RBF project regions to work harder to improve their vaccination coverage.

The present study found a reduction in urban–rural vaccination inequalities attributable to the RBF intervention, unlike a study found in Canada [30]. That study reported that a pay-for-performance system maintained socioeconomic inequalities in vaccination rates, but it did not reduce the pre-existing inequalities between social groups [30]. Pay for performance models focusing on the supply side could improve the quality of care received by disadvantaged groups, but it may not increase their likelihood of seeking services [39]. The immunization indicators remunerated in The Gambia are on the supply side of the pay for performance scheme meaning that the burden to increase the number of services delivered is on health workers. One of the main activities health workers can engage in to improve coverage is tracing vaccination defaulters. Defaulting (sometimes referred to as dropping out) is the main driver for suboptimal coverage in The Gambia, as illustrated by the lower coverage for later than earlier doses in the national schedule [40]. By definition, a defaulter is a child who received an earlier vaccine dose but not a later dose that is due. In that case, it would still be a matter of tracing children with access to vaccination and their caregivers were at least initially willing to bring for vaccination. Health workers in RBF regions may be more motivated to trace defaulters to improve their performance, thereby potentially narrowing the rural–urban full vaccination gap as pay-for-performance schemes can encourage health workers to improve their performance [41].

### 4.1 Strengths and limitations

This study used datasets from two nationally representative surveys to assess vaccination and equity following RBF implementation in The Gambia. The same institutions implemented the surveys using the same methodology, including sampling techniques and similar interview questions. This enhanced comparability of survey results of the two periods and favoured the use of more robust analytical methods such as the difference-in-differences approach used. The high vaccination card retention rates (at least 90% in each survey) minimized the chance for recall bias.

Though we used a difference-in-differences study design, we would like to bring the attention of readers the cross-sectional nature of the data when interpreting our findings. Generally, our sample size appears large, but it may be small, especially for the rural–urban inequality analysis resulting to lower power and consequently increasing the chance for a type 2 error. Moreover, as evident from the unbalanced comparison groups, particularly when considering the urban–rural distribution, selection bias could potentially threaten the validity of the findings. The RBF project was implemented in selected regions that were more rural and poorer. Perhaps, if the project was rolled out nationally, we would have detected its impact on vaccination coverage.

Another caveat that could influence our results is the gradual implementation of an electronic immunization register in two non-RBF health regions in the second quarter of 2017. The electronic register generates a defaulters' list health workers can use to trace children who have not returned for their due vaccine doses. Health workers received no extra motivation to use the list. Up to June 2018, it was used only in four of the total forty health facilities offering immunization services in those regions. Enrolment of health facilities in two regions was completed in January 2021.

### 4.2 Potential policy and research implications

Our study has contributed to the literature on the impact of pay for performance programs on health service delivery by shedding light on the potential effects of the RBF scheme on vaccination coverage and equity. The evidence from this study suggests that the pay for performance approach used may not be sufficient in addressing vaccination coverage challenges but could improve rural–urban disparities. It also showed that traditionally poor performing areas in vaccination could pick up, although the reasons for this are beyond the scope of this study. Future studies may attempt to identify why non-RBF areas had higher improvements in coverage and also evaluate the impact of RBF on inequalities in other health outcomes to better guide RBF implementation strategies in the country. Possibly, the RBF approach should be modified to be equity-focused and address vaccination-related demand-side barriers at the community level. Concentrating on the supply side of service delivery does not seem to lead to meaningful improvements in vaccination coverage. Full vaccination is just one of the many indicators for which the impact of the RBF project can be evaluated. It will be necessary for future studies to examine the impact of the project on service utilization and equity of other indicators to inform policy better. A more detailed evaluation focusing on whom and how the RBF project worked will shed more light on understanding the project.

## 5 Conclusions

We found that, on average, vaccination coverage improved over the study period. However, improvement in vaccination coverage was surprisingly lower in RBF than non-RBF intervention areas, but rural-favoured inequalities decreased more in the RBF regions post implementation than control regions. In conclusion, our study failed to attribute the apparent improvement in national vaccination coverage to the RBF project but recognized its positive contribution to the improvement in rural-favoured disparities. The significant coverage gain observed signals a possibility to extend the benefits of vaccination to all children in The Gambia despite the existing challenges.

## Funding

This research did not receive any specific grant from funding agencies in the public, commercial, or not-for-profit sectors.

## CRediT authorship contribution statement

**Alieu Sowe:** Conceptualization, Formal analysis, Validation, Methodology, Writing – original draft, Writing – review & editing. **Fredinah Namatovu:** Conceptualization, Validation, Methodology, Formal analysis, Writing – review & editing. **Bai Cham:** Conceptualization, Methodology, Validation, Formal analysis, Writing – review & editing. **Per E. Gustafsson:** Conceptualization, Methodology, Validation, Formal analysis, Writing – review & editing.

## Declaration of Competing Interest

The authors declare that they have no known competing financial interests or personal relationships that could have appeared to influence the work reported in this paper.
