# Supplementary material for: Does a pay-for-performance health service model improve overall and rural–urban inequity in vaccination rates? A difference-in-differences analysis from the Gambia
Source: Vaccine X. 2022 Aug 17;12:100206. doi: 10.1016/j.jvacx.2022.100206 (PMC9424534; doi:10.1016/j.jvacx.2022.100206)
Supplement: Supplementary data 1 [file mmc1.docx]

* Paper title: Does a pay-for-performance health service model improve overall and rural-urban inequity in vaccination rates? A difference-in-difference analysis from The Gambia

* Authors

* Alieu Sowe, Fredinah Namatovu1, Bai Cham, and Per E. Gustafsson

* Correspondence: alieu.sowe@umu.se / sowea1@yahoo.com

clear all

//version 17

set more off

cd "C:\Users\hp\Desktop\DHS datasets"

use "gmkr60dt\GMKR60FL.DTA", clear

* Log results

capture log close

log using "XXXX", text replace

* Create treatment and time variables

generate yr = 2013

compress // compress dataset to save memory

save 2013, replace // save 2013 dataset

* Call in 2019/2020 dataset

use "GMKR81DT\GMKR81FL.DTA", clear

generate yr = 2020

compress // compress dataset to save memory

save 2020, replace // save 2019/2020 dataset

* Append 2013 dataset

append using 2013

label variable yr "Year"

keep if b8 == 1 // keep children 12 - 23 months age range

save "Working dataset.dta", replace // save full dataset

* Keep those with vaccination cards seen for sensitivy analysis

*keep if h1 == 1

* Loal government area variable

rename v024 lga

label variable lga "Local Government Area"

* To generate a variable containing RBF implementation status

generate rbf = 0

replace rbf = 1 if (lga != 1 & lga != 2 & lga != 3) & yr == 2020

label define yn 0 "No" 1 "Yes"

label values rbf yn

label variable rbf "RBF implemented"

* To generate a variable differentiating regions in which RBF was implemented from those in which it was not implemente.

generate rbflga = 0

replace rbflga = 1 if (lga != 1 & lga != 2 & lga != 3)

label define iy 0 "Non-RBF" 1 "RBF"

label values rbflga iy

label variable rbflga "RBF status"

* To indicate the year RBF was implemented

generate rbfyr = 0

replace rbfyr = 1 if yr == 2020

label values rbfyr iy

* Tabulate rbf and yr and check missingness

tab rbf, m

tab yr, m

* Residence variable

recode v025 (1 = 0 "Urban") (2 = 1 "Rural"), generate (residence)

label variable residence "Residence"

* Weighting variables

gen wgt = v005/1000000

label var wgt "Survey weights"

rename v021 psu

rename v022 strata

* To create full vaccination variable using the necessary vaccine doses

*To see minimum and maximum values of variables

tabstat h2 h3 h4 h5 h6 h7 h8 h9, stat(min max)

*To tabulate variables including missing values

foreach h in h2 h3 h4 h5 h6 h7 h8 h9 {

tab `h', m

}

*To know how values were labelled

foreach h in h2 h3 h4 h5 h6 h7 h8 h9 {

tab `h', m nol

}

*To recode the variables and tabulate them

foreach h in h2 h3 h4 h5 h6 h7 h8 h9 {

recode `h' (0 = 0 "No") (1/3 = 1 "Yes") (8/. =.), gen (r`h')

tab r`h', m

tab r`h', m nol

}

*To label and rename variables

label variable rh2 "Received BCG"

ren rh2 bcg

label variable rh3 "Received Penta 1"

ren rh3 penta1

label variable rh5 "Received Penta 2"

ren rh5 penta2

label variable rh7 "Received Penta 3"

ren rh7 penta3

label variable rh4 "Received Polio 1"

ren rh4 opv1

label variable rh6 "Received Polio 2"

ren rh6 opv2

label variable rh8 "Received Polio 3"

ren rh8 opv3

label variable rh9 "Received Measles"

ren rh9 measles

*To generate the final outcome variable (receiving BCG, measles, & doses each of penta and polio)

gen fulvac1 = 0

replace fulvac1 = 1 if (bcg & penta1 & penta2 & penta3 & opv1 & opv2 & opv3 ///

& measles) == 1

replace fulvac1 = 0 if (bcg & penta1 & penta2 & penta3 & opv1 & opv2 & opv3 ///

& measles) != 1

tab fulvac1, mis

recode fulvac1 (0 . = 0 "No") (1 = 1 "Yes"), gen (fulvac)

label variable fulvac "Fully vaccinated"

tab fulvac, mis

tab fulvac1 fulvac

*Mathers' age

tab v013, m

tab v013, m nol

recode v013 (1 2 = 1 "15-24 years") (3 = 2 "25-29 years") (4 = 3 "30-34 years") (5 6 7 = 4 "35-49 years"), gen (agegr)

tab agegr

label variable agegr "Mother's age group"

tab age, m

tab bord, m

tab bord, m nol

recode bord (1 = 1 "1") (2 3 = 2 "2 - 3") (4 5 = 3 "4 - 5") (6/15 = 4 "6 & +"), gen (bon)

label variable bon "Birth order"

tab bon

*Wealth

recode v190 (1/2 = 1 "Poor") (3 = 2 "Middle") (4/5 = 3 "Rich"), generate (wealth)

label variable wealth "Household Wealth"

*Occupation

recode v717 (0 6 96 99 = 0 "Not working") (1 2 5 8 10 3 4 7 9 = 1 "Working"), gen (occupation)

label variable occupation "Occupation"

tab occupation

*Mother's education

recode v106 (0 = 0 "No education") (1 = 1 "Primary") (2 3 = 2 "Secondary & above"), gen (education)

label variable education "Maternal education"

*Is distance to the health center a problem

recode v467d (1 9 = 1 "Big problem") (2 = 0 "Not a big problem"), ///

gen (distance)

label variable distance "Distance to HF"

*Ethnicity

recode v131 (1 = 1 "Mandinka") (2 6 = 2 "Wolof") (4 = 3 "Fula") (3 5 6 = 5 "Others") (6/96 996 97 997 999 = 6 "Non-Gambians"), gen (ethnicity)

label variable ethnicity "Ethnicity"

tab ethnicity, m

*Marital status

recode v501(1 2 = 1 "Currently married") (0 3 4 5 = 0 "Not currently married"), ///

gen (marstat)

label var marstat "Marital Status"

*Child's sex

rename b4 sex

label variable sex "Child's sex"

* Child's age

rename v012 age

label variable age "Age"

* Prepare Stata to analyse survey data

svyset psu [pweight=wgt], strata(strata)

* Table 1

* Descripitive and bivariate analysis (activate code and select year as necessary in the loop)

local sarjo residence wealth marstat sex ethnicity education occupation distance agegr bon

foreach a of local sarjo {

svy: tab `a' rbflga if yr == 2013, row count // 2020

*svy: glm fulvac i.`a', family(poisson) link(log) eform

}

* Table 2

svy: proportion fulvac, over(yr)

svy: proportion fulvac, over(rbflga)

svy: proportion fulvac, over(residence)

svy: proportion fulvac if residence == 0, over(yr) // Urban areas

svy: proportion fulvac if residence == 1, over(yr) //Rural areas

svy: proportion fulvac if rbflga == 0, over(yr) // Non-RBF LGAs

svy: proportion fulvac if rbflga == 1, over(yr) //RBF LGAs

svy: proportion fulvac if residence == 0 & yr == 2013, over(rbflga)

svy: proportion fulvac if residence == 1 & yr == 2013, over(rbflga)

svy: proportion fulvac if residence == 0 & yr == 2020, over(rbflga)

svy: proportion fulvac if residence == 1 & yr == 2020, over(rbflga)

svy: proportion fulvac if residence == 0, over(rbflga)

svy: proportion fulvac if residence == 1, over(rbflga)

* Create a global macro to store the covariates for the adjusted regression models

global yero i.wealth i.marstat i.sex i.ethnicity i.education i.occupation i.distance i.agegr i.bon

* Main analysis

* TOTAL COVERAGE - Table 3

svy: glm fulvac i.yr, family(poisson) link(log) eform

svy: glm fulvac i.yr i.residence $yero, family(poisson) link(log) eform

* RBF IMPLEMENTATION (DiD)

gen did = rbflga*rbfyr

* Difference-in-Differences

svy: glm fulvac did i.rbflga i.yr, family(poisson) link(log) eform

svy: glm fulvac did i.rbflga i.yr i.residence $yero, family(poisson) link(log) eform

* RBF IMPLEMENTATION AND RESIDENCE TYPE - DiDiD

gen didid = rbflga*rbfyr*residence

* Difference-in-Differences-in-Differences

svy: glm fulvac i.rbflga i.yr i.residence, family(poisson) link(log) eform

svy: glm fulvac i.rbflga i.yr i.residence i.rbflga#i.yr#i.residence $yero, family(poisson) link(log) eform

* Figure 1: Adjusted predicted mean vaccination coverage margins for rural and urban areas in 2013 and 2020 by RBF implementation status.

svy: glm fulvac i.rbflga i.yr i.residence i.rbflga#i.yr#i.residence $yero, family(poisson) link(log) eform

margins i.yr i.residence i.yr#i.residence, over(rbflga) citype(wilson)

* END
