# Supplementary material for: Does a pay-for-performance health service model improve overall and rural–urban inequity in vaccination rates? A difference-in-differences analysis from the Gambia
Source: Vaccine X. 2022 Aug 17;12:100206. doi: 10.1016/j.jvacx.2022.100206 (PMC9424534; doi:10.1016/j.jvacx.2022.100206)
Supplement: Supplementary data 3 [file mmc3.docx]

**Table 1:** Weighted crude prevalence rate ratios of the change in vaccination coverage in The Gambia 2013 – 2020 excluding vaccination history by recall.

**Table 2:** Weighted adjusted prevalence rate ratios of the change in vaccination coverage in The Gambia 2013 – 2020 excluding vaccination history by recall.

**Table 3:** Weighted crude Difference-in-Differences analysis of full vaccination coverage between RBF- and non-RBF intervention areas 2013 - 2020 excluding vaccination history by recall.

**rbflga:** A binary variable denoting RBF implementation status (= 1 if region implemented RBF and 0 if otherwise)

**Table 4:** Weighted adjusted Difference-in-Differences analysis of full vaccination coverage between RBF- and non-RBF intervention areas 2013 - 2020 excluding vaccination history by recall.

**rbflga:** A binary variable denoting RBF implementation status (= 1 if region implemented RBF and 0 if otherwise)

**Table 5:** Weighted crude Difference-in-Differences-in-Differences analysis of full vaccination coverage between urban and rural areas in RBF- and non-RBF intervention regions 2013 - 2020 excluding vaccination history by recall.

**rbflga:** A binary variable denoting RBF implementation status (= 1 if region implemented RBF and 0 if otherwise)

**Table 6:** Weighted adjusted Difference-in-Differences-in-Differences analysis of full vaccination coverage between urban and rural areas in RBF- and non-RBF intervention regions 2013 - 2020 excluding vaccination history by recall.

**rbflga:** A binary variable denoting RBF implementation status (= 1 if region implemented RBF and 0 if otherwise)
